# Rapid Quantitative Assessment of Residual Stress States in PLA Components Enabled by the Combination of Photoelasticity and the Hole Drilling Method

**DOI:** 10.1002/bip.70026

**Published:** 2025-05-31

**Authors:** Olzhas Tlegenov, Margarita Reit, Jan‐Christoph Zarges, Alexander Liehr, Thomas Niendorf, Hans‐Peter Heim

**Affiliations:** ^1^ Institute of Material Engineering, Metallic Materials University of Kassel Kassel Germany; ^2^ Institute of Material Engineering, Polymer Engineering University of Kassel Kassel Germany

**Keywords:** hole drilling method, injection molding, PLA, polariscope, residual stress

## Abstract

Poly(lactic acid) (PLA) is one of the most prominent biopolymers and is considered a viable alternative to petroleum‐based polymers. While it exhibits comparable properties to conventional polymers like PET, in certain applications, particularly those involving elevated temperatures, PLA has performance limitations. In addition, the properties of PLA are dependent on the processing parameters in injection molding. Non‐optimal process parameters can lead to defects or undesirable effects that cannot be detected immediately after injection molding. This includes orientation and residual stresses, which significantly influence the material and failure properties. The present study investigates the influence of injection molding machine settings on the residual stress state in PLA components. Test specimens were produced using two different mold tools: an ejector pin and a full‐surface ejector, while varying key machine settings. Residual stress was assessed using a polariscope and the hole drilling method. The polariscope identified distinct isochromatic fringe patterns, particularly near the sprue, indicating regions of elevated residual stress. The hole drilling method confirmed the presence of high residual stress at the specimen edges, extending to a depth of 600 μm, with a peak stress value of 47 MPa. Results revealed that the ejector pin mold induced both tensile and compressive stress states, whereas the full‐surface ejector mold predominantly caused high compressive stresses at the edges. These findings highlight the importance of optimizing injection molding parameters to minimize residual stress and improve the mechanical performance of PLA components.

## Graphical abstract

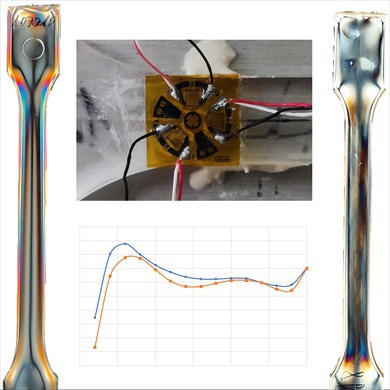

## 1 Introduction

The demand for environmentally sustainable and economically viable materials across diverse applications is increasing, driven by both societal consciousness and political imperatives. This aspect is particularly pronounced in the polymer industry. Here, a central theme revolves around substituting petroleum‐derived materials with bio‐based alternatives. These alternatives, derived from renewable sources or possessing biodegradable properties, are promising, as they are derived from renewable raw materials. Among them, poly(lactic acid) (PLA) emerges as a frontrunner, being produced entirely from renewable resources [1, 2]. PLA is one of the most prominent biopolymers and is considered a viable alternative to petroleum‐based polymers. It exhibits comparable properties to conventional polymers like PVC or PET [3, 4]. Due to its compatibility with the human body, PLA is widely used not only in medicine but also in the food and packaging sector and is a common filament material for 3D printing [5, 6]. However, in certain applications, particularly those involving elevated temperatures, PLA has performance limitations. The incorporation of additives, blending with other polymers, or the addition of fillers can enhance its properties and extend its applicability [3]. The applications mentioned are often disposable products or intended for short‐term use, to extend the application range of PLA or to improve existing properties according to specific requirements. Unfortunately, there is a gap in understanding the long‐term stability of PLA, which remains poorly understood in current materials science. Since these properties are already significantly affected by PLA processing, it is crucial to study the influence of production parameters on the characteristics of the material. This is the only way to reliably achieve stable material properties and eventually expand the use and applications of PLA.

The properties of components manufactured from thermoplastic polymers are predominantly influenced by fabrication conditions such as temperature, pressure, shear rate, and cooling rate, which affect the materials structure [7, 8, 9]. Many fundamental properties originate during the fabrication of the component. Thus, the primary objective of each manufacturer is to ensure the choice of optimal conditions throughout the manufacturing process, thereby facilitating the production of components that exhibit superior quality and align with the specific demands of the intended application. In the context of the present work, the manufacturing process of injection molding is examined in detail.

The injection molding process is a common process in which a huge number of components can be produced in a short time. Numerous machine settings can be adjusted on the injection molding machine, including parameters relating to temperatures, times, or pressure curves [10, 11]. Only when an optimal operating point has been found, that is, when the interaction of the machine settings is optimal, can components be produced that do not exhibit any defects. Defects in polymer components include, for example, unfilled cavities, shrinkage effects, or burrs on the edge of the component [12]. These types of defects are easy to recognize immediately after manufacture, which makes it easy to rectify the issue. The machine settings can be adjusted for this purpose. However, there are also defects or undesirable effects that cannot be detected immediately after injection molding. These include the warpage or twisting of components in case orientations or residual stress are introduced into the component as a result of the injection molding process [13]. Residual stress is defined as a stress component not induced by prevailing external loads but intrinsically present in components due to their manufacturing history [14]. Orientation is defined as the tendency of the spatial distribution of chain segments to show directions of preference [15]. Both orientations and residual stress states are not visible to the bare eye inspection after injection molding. However, due to the presence of both, components can bend or twist into an undesired shape after cooling and crystallization processes, respectively, have been fully accomplished. In addition to the undesired bending of the components, depending upon the field of application, residual stress can be released over the course of time, for example, at elevated temperatures, meaning that specified material properties are not maintained and, in turn, the service life is shortened [16, 17]. Since the origin and manifestation of the residual stress considered in this work is the manufacturing process, it is important to understand the relationship between the machine setting parameters and the resulting residual stress.

Table 1 lists the most influential process parameters on the morphology and associated residual stress states, according to literature research. These setting parameters, such as injection pressure or flow rate, play a critical role in determining the distribution and magnitude of residual stress within the material. The impact column outlines how each setting parameter specifically influences the materials properties, for instance by altering molecular orientation or relaxation, which in turn affects the stress patterns. Due to the complex nature of the elementary mechanisms involved, the table also includes sources that used numerical simulations to calculate residual stress states. While there may be additional parameters that influence the residual stress states, the process settings listed in Table 1 were the only ones that resulted in noticeable changes to the isochromatic fringe patterns of PLA during the course of the experiments (cf. Chapter 3). The table highlights how each parameter's influence was assessed and observed, either experimentally or through simulation, providing a clear link between the settings and the resulting stress state within the material.

**TABLE 1:** Influence of machine setting parameters on the residual stress state.

Setting parameter	Impact	Stress pattern
Injection pressure	Residual stress [18]	Edge: Tensile Core: Compressive
Injection flow rate (slow)	Increases pressure in edge region [18]	
Packing pressure (high)	Residual stress reduced [14, 18]	Edge: Tensile Core: Compressive
Cooling time (slow)	Ordered molecular orientation, residual stress reduced [19]	
Temperature difference melt and mold	Shrinkage causes residual stress, induced by post‐crystallization	
Mold temperature (low)	Prevention of relaxation promotes residual stress [20, 21]	Edge: Compressive Core: Tensile
Mold temperature (high)	Improved heat transfer Residual stress reduced [20, 21, 22]	

Residual stress in polymers mainly evolves due to the temperature difference between the outer wall and the core of components during injection molding [18]. Ideally, the melt should wet the outer wall of the cavity, solidify, and thus form the edge layer of the component, which is subsequently filled with the remaining melt that cools and solidifies with a delay. The different cooling and solidification conditions of the melt in the aforementioned areas cause a change in volume due to the solidification of the melt in the core to be hindered by the previously solidified edge areas [14]. This generates compressive stress in the edge areas and tensile stress in the core [18]. The overall residual stress state of a component is therefore affected by flow, cooling, and expansion compression [18].

Recent studies showed interest in residual stress and its influence on material properties; however, most of them only simulated evolution of residual stress during production [23] and measured it indirectly [24]. Quantitative values obtained by standardized experimental techniques are not provided. In the present study, the phenomenon of test specimen warping following the injection molding process was studied in depth, based on the combination of complementary techniques providing for rapid qualitative and reliable quantitative results. In order to comprehend the underlying reasons for the exhibited curvature of the test specimens, efforts were initially directed toward identifying a discernible pattern by photoelasticity, to be able to predict material behavior depending on the color distribution. There are many different residual stress‐measuring techniques; they can be destructive, partially destructive, or fully destructive methods. Two methodologies that can also be applied to polymers are frequently used to reliably determine residual stress states: X‐ray diffraction (XRD) in the case of (semi‐) crystalline materials [25] and the hole drilling method [26, 27, 28]. The XRD method is based on the diffraction of electromagnetic waves on lattice structures. The hole drilling method is based on the elastic behavior of the material. Induced residual stresses are released due to the drilling of a small hole in the form of displacement, which is acquired with the help of strain gauge rosettes. In the present case, the XRD‐based approach was thought not to be reliable due to the low amount of crystalline structures. Consequently, to conduct an in‐depth investigation of the test specimens and to assess the underlying residual stress states in a very efficient, rapid manner, the combination of a polariscope (photoelasticity, qualitative) and the hole drilling method (quantitative analysis) was chosen [13]. The polariscope, which exploits the effect of elasticity due to birefringence, allows stress to be calculated and is the only experimental method for rapidly visualizing residual stress states. The basic requirement is that the polymer is transparent so that polarized light can pass through the test specimen. The residual stress state is then determined qualitatively based on the birefringence. This is achieved by two crossed polarizing filters and a light source. The birefringence leads to isochromatic fringe patterns eventually representing the prevailing stress and the stress direction, respectively [29]. As a secondary method to investigate the properties of the test specimens, the hole drilling method was employed. The hole drilling method exploits the elastic behavior of the material upon its incremental removal [13]. Released strain is mainly measured using strain gauges. Finally, residual stresses are processed using an integral calculation method by calculating calibration matrices that have been provided by ASTM Norm [28, 30]. The application of this method requires precise knowledge of the specific polymer and its structural properties, such as crystallinity.

Test specimens with various residual stress distributions were fabricated via injection molding. This was accomplished by varying machine settings and utilization of two different mold tools for test specimen production, respectively. The resultant residual stress states in the test specimens were subsequently analyzed using a polariscope. Test specimens exhibiting different isochromatic fringe patterns were then selected for quantitative assessment by the hole drilling method. By quantitative assessment of residual stress in the test specimens, a correlation between residual stress and isochromatic fringe patterns could thus be established [29]. Literature often suggests that the polymer melt in the core cools down later than in the outer layers, leading to tensile stress inside and compressive stress on the surface [14, 18]. Due to the popular use of PLA in 3D printing, residual stresses have already been investigated using the drill hole method [31]. However, a detailed examination of residual stresses in PLA in larger component geometries and varying parameters during injection molding has never been carried out to such an extent to prove this assumption.

Within the scope of the present study, the synergy of isochromatic fringe pattern analysis for rapid localization of regions with induced residual stress and the hole drilling method for quantitative determination of stress distribution for PLA was researched, aiming to contribute to the utilization of bio‐based materials and showcase the possibilities of material characterization for future structural construction purposes.

## 2 Materials and Methods

### 2.1 Materials

For the investigations of residual stress, semi‐crystalline PLA Luminy L130 was used, which was provided by TotalEnergies Corbion (NS Gorinchem, the Netherlands). Table 2 lists the most important thermal and mechanical properties of PLA Luminy L130 [33].

**TABLE 2:** Thermal and mechanical properties of the examined PLA Luminy L130 [32].

Material property	Value
Density	1.24 g/cm3
Melt flow index	23 g/10 min
Melting temperature	175°C
Glass transition temperature	60°C
Tensile modulus	3500 MPa
Tensile stress	50 MPa
Elongation at break	5 (%)

### 2.2 Injection Molding

To fabricate the type 1A test specimen according to DIN EN ISO 527‐2, a hydraulic injection molding machine Allrounder 320C Golden Edition (Arburg GmbH + Co. KG, Lossburg, Germany) with a clamping force of 50 kN was used.

**FIGURE 1:**
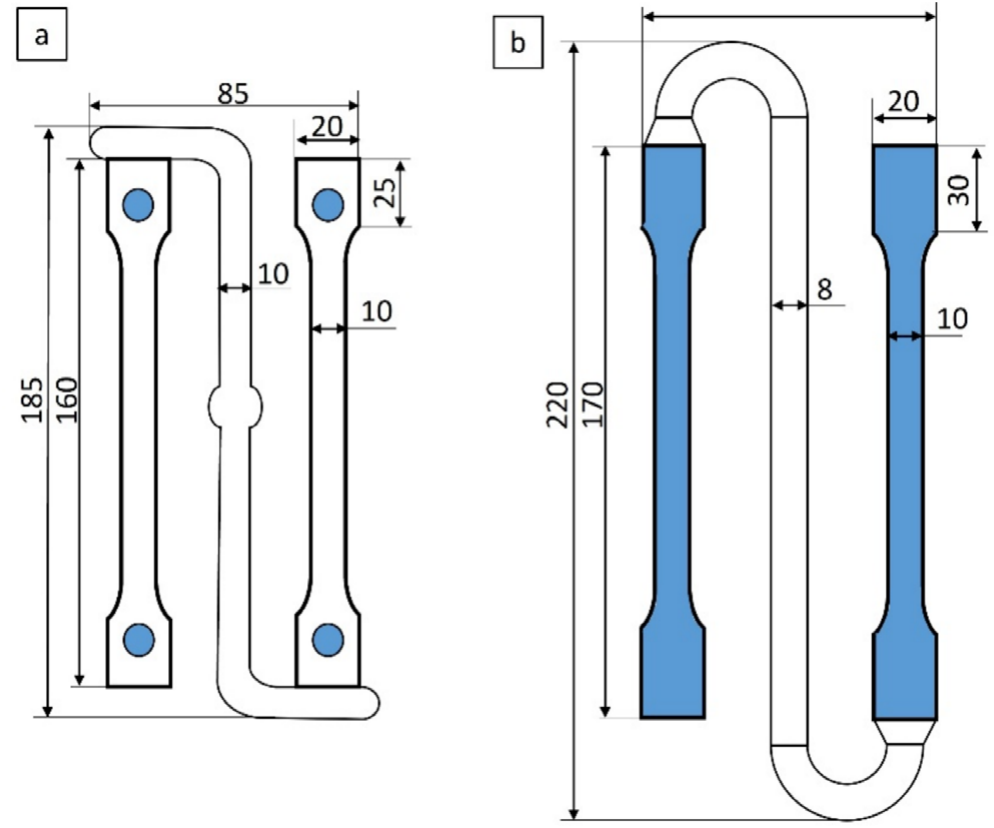
Both evaluated mold tools with ejector pins (a, mold 1) and full‐surface ejectors (b, mold 2) highlighted in blue included with important dimensions.

Standard injection mold 1 with ejector pins used at the IfW was initially employed for this purpose (see Figure 1a). As the aim of this paper was to deliberately produce different structures and the resulting residual stresses, the process parameters were varied over a very wide range (see Table 3). When carrying out the injection molding tests with the different process variations using mold 1, it was found that the test specimens were only automatically demoldable with variations 1 and 2. In the case of variations 3, 4, and 5, the test specimens were not ejected due to their flexibility but were considerably deformed by the ejector pins as well as due to the manual removal from the mold. These deformations and the associated stresses in the test specimen did not allow a differentiated consideration of the residual stresses in the test specimen, which is why the test specimens from mold 1 and variations 3, 4, and 5 were not considered in the further tests.

**TABLE 3:** Injection molding parameters for both used mold tools.

Parameter	Mold 1					Mold 2	
	Variation 1	Variation 2	Variation 3	Variation 4	Variation 5	Variation 6	Variation 7
Injection pressure (bar)	630	630	670	630	630	1000	1000
Injection flow (cm^3^/s)	30	30	45	30	30	30	30
Packing pressure (bar)	700	700	700	850	700	950	950
Cooling time (s)	45	45	45	45	25	30	15
Mold temperature (°C)	30	50	30	30	30	30	30

**FIGURE 2:**
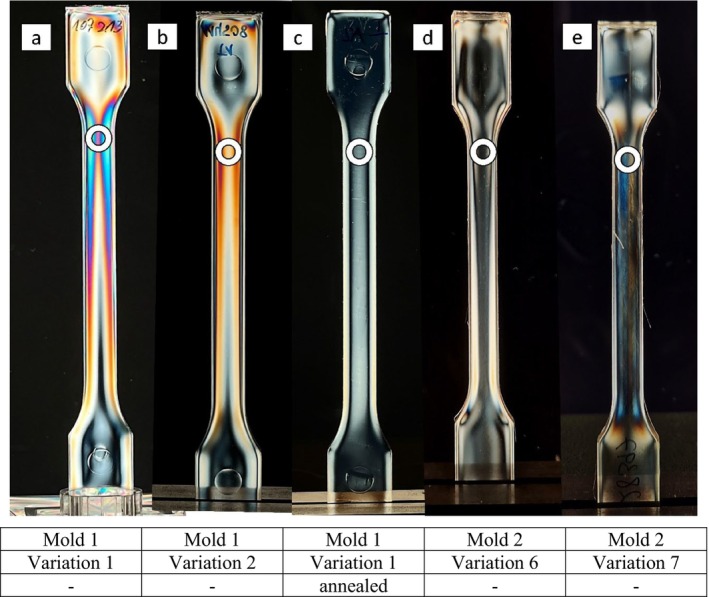
Illustration of the isochromatic fringe patterns of all test specimens that were measured using the hole drilling method. The measuring point is marked with a white circle. The table shows the manufacturing variations a to e described in Table 3.

However, in order to be able to consider the influence of further process variations in injection molding, mold 2 with a surface ejector plate was used. The surface ejector significantly reduced the deformation of the test specimens during ejection and thus also reduced the stresses induced in the test specimen by the ejection process (see Figure 2d,e). When switching to mold 2, not all settings of mold 1 could be adapted in the same way. In order to produce the same test specimen in terms of quality as with mold tool 1, the injection pressure as well as packing pressure were increased and the cooling time decreased (see Table 3).

Figure 1 shows the dimensions and the difference between the two mold tools used. Both specimens have a thickness of 4 mm, while mold 1 (left) has four punctual ejector pins (blue) in the area of the test specimen and mold 2 (right) and, on the other hand, has an ejector plate over the entire sample surface (blue). At least five test specimens of each variation were manufactured to allow comprehensive analysis.

Prior to the processing, the PLA was dried for 6 h at 100°C. The process parameters used are described in more detail in the following chapters, as they were varied for test purposes.

In order to measure test specimens with potentially higher residual stress as well as those with lower stress, a test specimen of variant 1 was annealed with the aim of reducing existing stresses (Figure 2c). Annealing the test specimens results in stress in the specimen being relieved, which is caused by the relaxation of the material [34]. The annealing or tempering itself was carried out between the melting point and the glass transition temperature [35]. This process involved examining PLA tempering at 60°C for 60 min.

### 2.3 Tensile Test

To investigate the tensile properties of the specimen as well as the Young's modulus for the calculation of the residual stress, tensile tests were carried out using the universal testing machine Z010 by Zwick Roell (Ulm, Germany) according to DIN EN ISO 527 at a speed of 5 mm/min. A tactile displacement sensor was used for the strain measurement.

**FIGURE 3:**
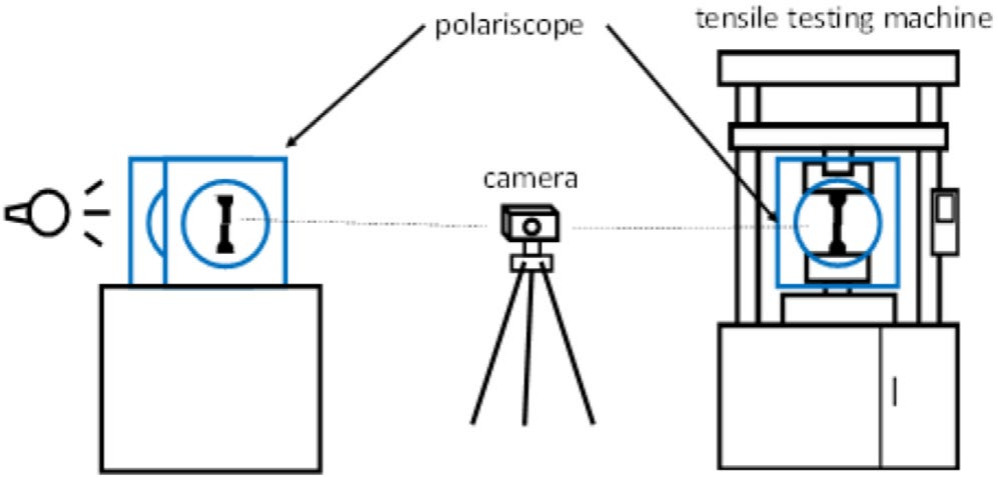
Schematic illustration of the use of the polariscope. The static test is shown, as is the setup of the polariscope in the tensile testing machine, with the use of which the test specimens were examined for isochromatic fringe patterns.

To examine how stress changes under load and to pinpoint areas of the highest local stress, in situ experiments were conducted. For this purpose, the tensile testing machine (Figure 3) was equipped with a polariscope, maintaining the polariscope settings to ensure comparability of isochromatic fringe patterns within the results of this study.

### 2.4 Differential Scanning Calorimetry (DSC)

**FIGURE 4:**
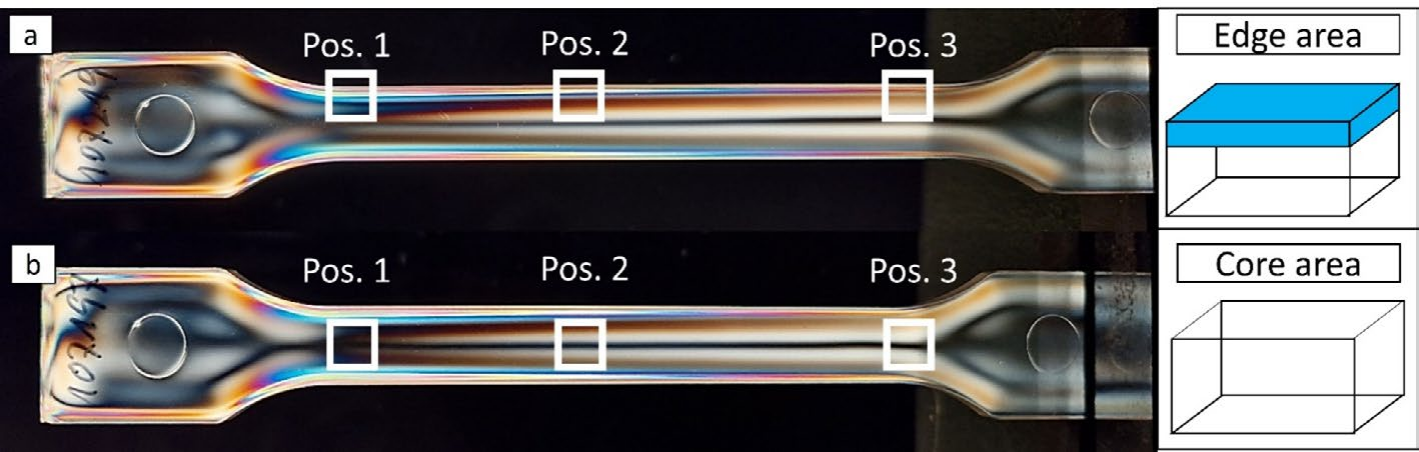
Location of DSC measurements marked with white squares and the semantical location of amorphous (a: edge area in blue, b: corearea) regions in a measurement sample (here mold 1, variation 1).

DSC measurements were carried out to investigate the crystallinity of different areas respectively for test specimens produced by both mold tools. For this purpose, samples with the dimensions of approximately 1 × 6 mm^2^ were cut out of the test specimens at three locations (see Figure 4): near the sprue (Pos. 1), in the middle (Pos. 2), and far from the sprue (Pos. 3) as well as from the edge and core area of the samples used.

For the crystallinity analyses, DSC measurements according to DIN EN ISO 11357 were carried out using the DSC module Q2000 (TA Instruments, New Castle, USA).

Two heating cycles were carried out with a cooling cycle in between, whereby the crystallization enthalpy was determined during the second heating cycle. Here, a constant heating rate of 10 K/min (sample weight approx. 10 mg) and a nitrogen atmosphere with a temperature program of 0°C–250°C were used. Regarding the following equation, the degree of crystallization X_c_ [%] was calculated using the enthalpy of fusion (∆H_f_), the enthalpy of crystallization (∆H_c_), and the mass fraction of the matrix (w). A value of 93.6 J/g (∆H_f_^0^) for 100% crystallinity of PLA was used.

(1) $$X_{c}\left[\%\right]=\left(\left(∆H_{f}-∆H_{c}\right)/w∆H_{f^{0}}\right)*100$$

### 2.5 Polariscope

The study utilized a diffuse light polariscope, type BS, manufactured by Tiedemann Instruments GmbH & Co. KG (Garmisch‐Partenkirchen, Germany). This device is equipped with a light box containing a white monochromatic sodium bulb as a light source, ensuring stable and uniform illumination and operating within a compensation analyzer. Key components include a linear polarizing filter, a rotating analyzer for generating and analyzing polarized light, and quarter wave plates. The polariscope allows for precise measurement of birefringence in polymer materials, which is essential for assessing mechanical stress and structural changes. Samples were securely positioned in a sample holder to guarantee even illumination. The images and videos were taken with a Nikon D3200 and a DX SWM VR ED IF Aspherical ⌀67 lens.

To examine the residual stress distribution in the manufactured test specimens, a polariscope was employed. Care was taken to ensure consistent polariscope settings in all images throughout this study, specifically referring to the positions of the polarizer and analyzer. The polarizer was in the starting position 0° and the analyzer was shifted by 90° to it, at a distance of 25 cm from each other.

The schematic diagram in Figure 3 shows how the polariscope was applied. On the one hand, the polariscope was used for static tests to localize the distribution of stress within the specimen. On the other hand, the polariscope was installed in the tensile testing machine in order to observe the color change with increasing load. The exposure time was 1/8 s and an increment of 5 s.

### 2.6 Hole Drilling Method

**FIGURE 5:**
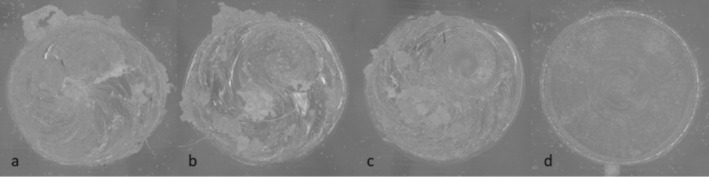
Different drilling techniques and settings for the air turbine—(a) to (c) air pressure from 3 to 1 bar, orbital drilling technique (1 mm drill), and (d) hand drilling (1 rps, 2 mm drill).

Residual stress analysis was performed according to ASTM E837‐20 [30] using the RS‐200 Residual Stress Milling Guide from Micro‐Measurements (Micro‐Measurements, Wendell, NC 27591, USA). It is critical to address the choice of material removal technique. As it was mentioned in [36], a different approach could lead to plastic deformation during drilling. Even if orbital drilling with an air turbine at 3 bar of air pressure is preferable due to the good chip removal and better hole geometry, the low thermal conductivity of PLA leads to low heat dissipation, which does not allow for proper hole geometry even with low settings of air pressure down to 1 bar (Figure 5). A hand drill was used to drill the hole at a speed of approximately one revolution per second to avoid additional influence on the polymer. To achieve a hole diameter of 2 mm, a 2 mm drill with a TiAlN coating (GARANT Diabolo VHM‐Microfräser) was used.

**FIGURE 6:**
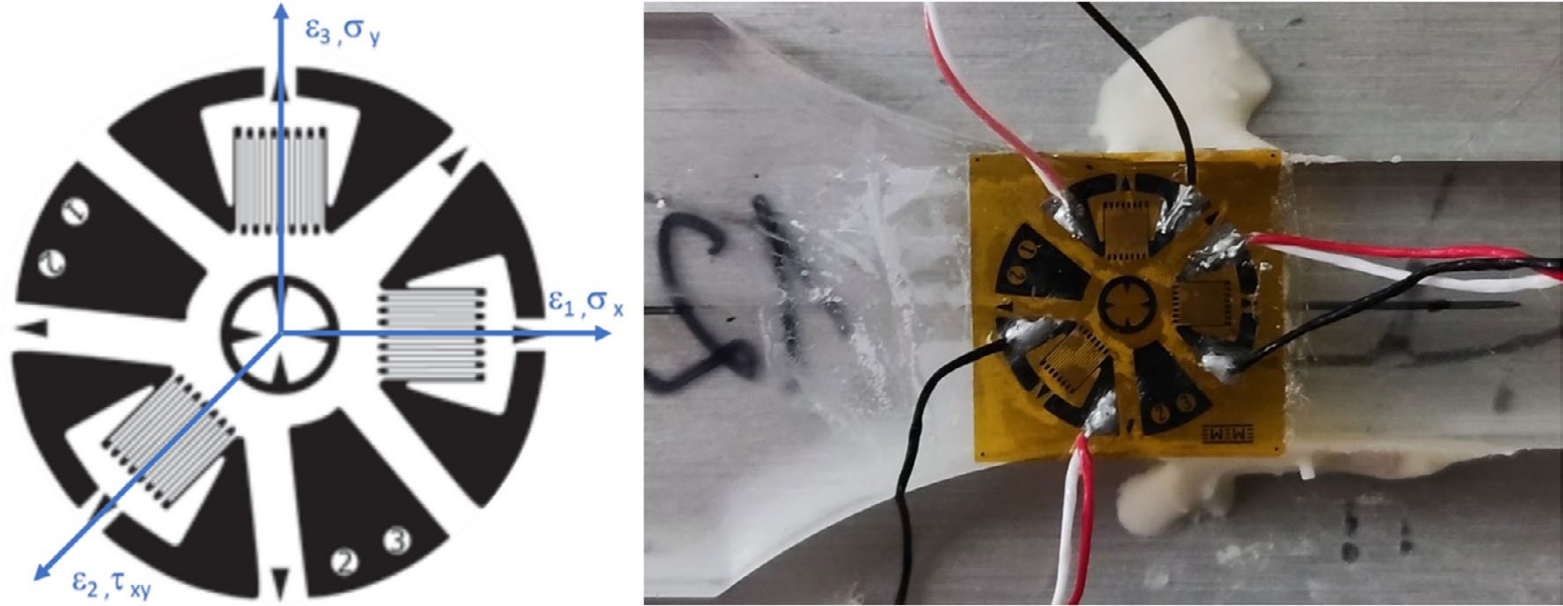
Applied strain gauge ES‐13‐062RE‐120 with measuring strain and calculated residual stress axes [30].

For specimen preparation, the entire part was glued to an aluminum plate to secure stability during the experiment (Figure 6). Ethanol was used to obtain a clean surface without damaging the specimen. To induce high surface energy and to prepare the surface for an adhesive connection between strain gauge and specimen, a primer (LOCTITE SF 770) was applied onto the measuring area. For strain measurement, strain gauges EA‐13‐062RE‐120 from Micro‐Measurement were used and connected to the amplifier Picas from Peekel Instruments (Peekel Instruments GmbH, Bochum, Germany) with a feed voltage of 0.5 V to reduce thermal interference [36]. The calibration matrices for strain–stress transformation were calculated using a polynomial procedure provided in the ASTM standard [30].

In line with the ASTM standard [30], it is crucial to test the material, in this case PLA, with respect to its mechanical isotropy. Anisotropy has a significant influence on the measurement and calculation process. To provide for an estimate of the degree of (an)isotropy of the PLA, a tensile test could be applied; however, due to the geometry restrictions in the present work, a compression test was chosen. This test comprised compression loading of the material in longitudinal and transverse directions to compare the stress‐displacement response directly (this procedure being thought to be sufficient to assess isotropy in present work). For this purpose, a compression test using a Kammrath & Weiss test rig (Kammrath & Weiss GmbH, Schwerte, Germany) at a strain rate of 1 μm/s was conducted. Specimens with nominal dimensions of 10 × 10 × 4 mm^3^ were cut from the initial specimens (of both molding tools) using a water‐cooled diamond wire saw to ensure minimum influence on the properties of the material being tested.

## 3 Results

### 3.1 Polariscope

In Figure 2, the stresses in the specimens produced with different variations of the injection molding parameters or annealing states are shown. Here, the test specimen produced with process variation 1, depicted in Figure 2a, shows significant isochromatic fringe patterns near the sprue (section in which the melt enters), situated on the top of the image. The color intensity diminishes toward the side opposite to the sprue.

Figure 2b shows the test specimen from mold 1, variation 2 and it can be seen that the isochromatic fringe patterns in this test specimen are not as pronounced as in variation 1.

The result of the residual stress distribution after annealing is depicted in Figure 2c. It can be seen that all colors in the test specimen have disappeared under polarized light and the test specimen is completely transparent, which means that there are no or only very low stresses in the specimen. As mentioned in Section 3.1, the full‐surface ejector mold tool produced test specimens with very few to no isochromatic fringe patterns. Figure 2d shows one such test specimen, produced with mold 2 and process variation 6, which exhibits almost no isochromatic fringe patterns, while Figure 2e depicts a specimen of variation 7. In this test specimen, slight isochromatic fringe patterns can be observed, although they do not appear as symmetrically as in the test specimen with the ejector pin.

Throughout all tests conducted in the present study, intensified colors consistently appeared in the vicinity of the sprue. Therefore, in the subsequent examinations using the hole drilling method, this area was always selected for stress measurement, corresponding to the white circles in Figure 2.

### 3.2 In Situ Tensile and Bending Test

**FIGURE 7:**
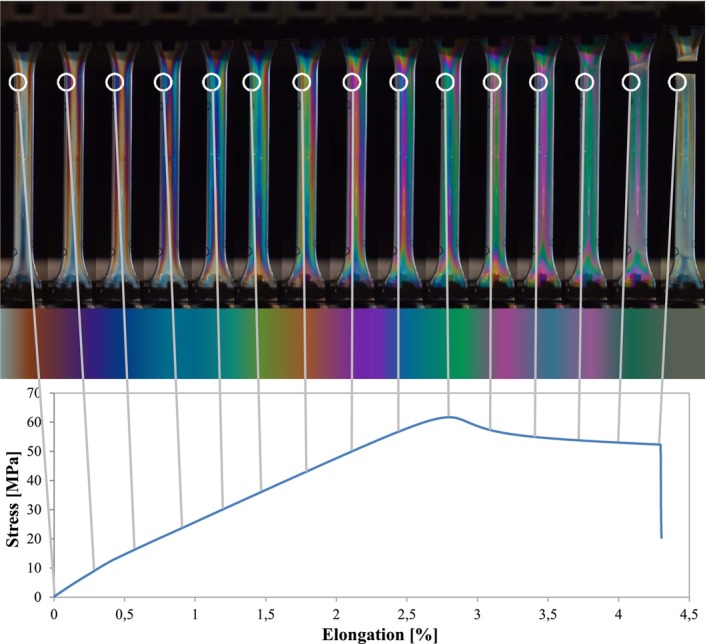
In situ tensile test in the polariscope and the corresponding Michel–Levy color chart of a test specimen produced with mold 1 and variation 2.

Figure 7 depicts the in situ tensile test, which was conducted at a constant test speed of 5 mm/min and the use of the polariscope. The entire tensile test was recorded using the video camera. Figure 7 shows images at intervals of around 20 s, documenting the progression of the test specimen from the unloaded state to failure. The total test duration was 120 s. A test specimen of mold 1 variation 2 was examined for the tests. These investigations aimed to document and localize isochromatic fringe pattern development and to analyze stress distribution under external load. It is evident that with increasing load, a change in color occurs, which repeats cyclically. This indicates increasing stress, which is reflected in the form of more intense colors meaning a structural change in the material, which influences the birefringence of the test specimen. A detailed analysis of color changes is presented in Figure 7, indicating the selected area (white circle) for color change. Initially, either no colors or clear colors up to a dark red are visible in the unloaded specimen. Subsequently, colors corresponding to the Michel‐Levy color chart appear [37]. The photoelastic behavior of our examined PLA is therefore consistent with the literature. With increasing load, the colors from this color chart cycle between green and pink. With each repetition, the colors become paler until the test specimen appears almost white. From an order number of 4, the colors are hardly recognizable. This order number represents a complete wavelength delay or repetition of a color [37]. The color repetitions originate from the vicinity of the sprue. From the sprue area, the colors develop almost symmetrically to the left and right. This color change repeats across the entire length of the specimen. After the specimen narrows in the area with the most pronounced colors, failure occurs, leading to fracture. From this investigation, a correlation has been established indicating that component failure occurs in areas where the highest amount of isochromatic fringe patterns corresponds to the highest residual stress.

**FIGURE 8:**
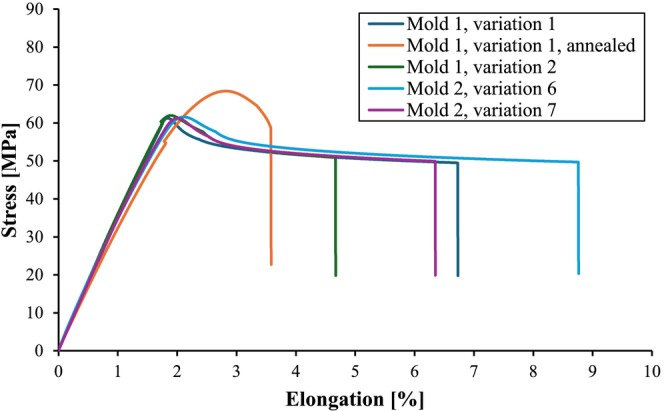
Stress–elongation curves as a result of tensile tests carried out on the specimen produced with the different mold tools and process variations.

In addition to the in situ tensile tests shown in Figure 7, tensile tests were carried out on all five batches of test specimens characterized here. The results in the form of a stress–strain diagram are shown in Figure 8.

It is initially clear that, apart from the annealed specimen, all other specimens show very similar curves as well as tensile strengths and only the elongation at break varies. In comparison, the annealed sample (mold, var. 1, annealed) shows the highest tensile strength and a significantly reduced elongation at break. From this, it can be deduced that the higher mold temperature (mold 1, variation 2) and when annealing the sample from variation 1 (mold 1, variation 1, annealed) lead either to a higher crystallinity or to a reduction in compressive stresses, which counteract the external force in the tensile test. The test specimens produced at the lower mold temperature of 30°C (mold 1, variant 1; mold 2, variant 6 and mold 2, variant 7) show the highest elongation at break, which may be due to low residual stresses or a low degree of crystallization.

**FIGURE 9:**
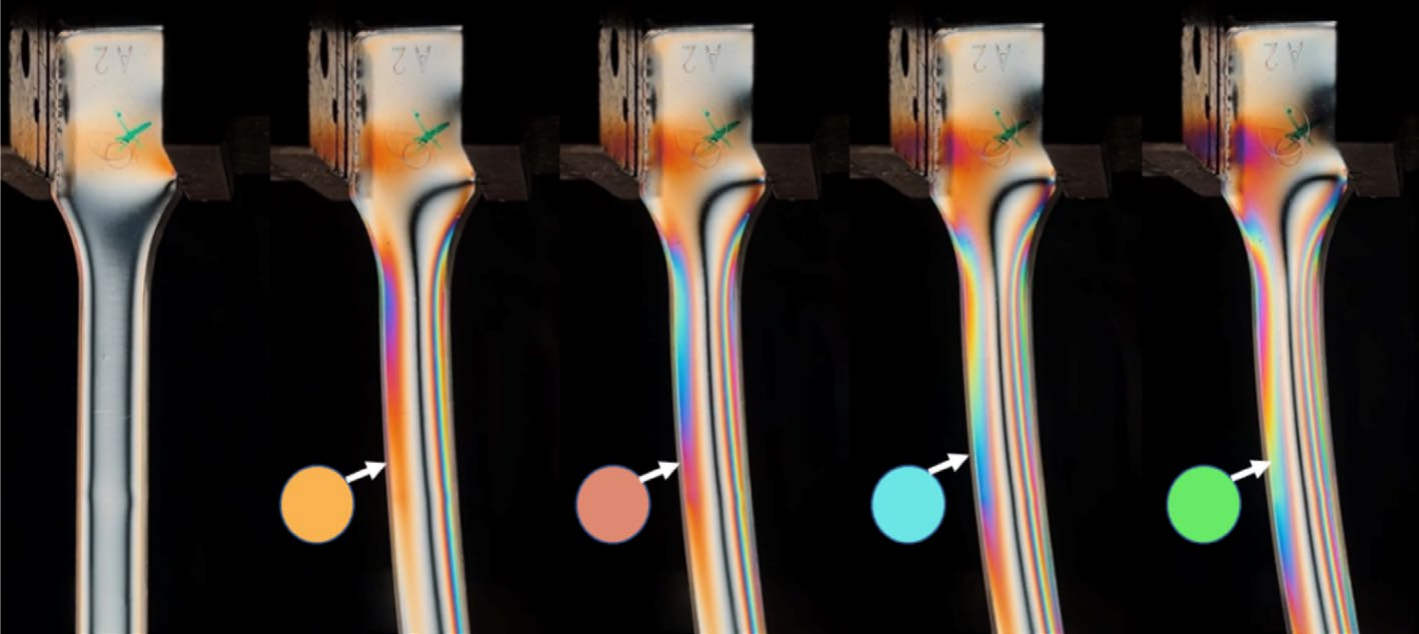
Annealed test specimen produced with the ejector pin mold tool (mold 1, variation 1) with less birefringence effects under bending load.

To make statements about the effects of external load on the test specimen with a small amount of isochromatic fringe patterns on stress distribution, a bending test of a tempered specimen is depicted in Figure 9. In contrast to the tensile test, the bending test was chosen in order to visualize both tensile and compressive loads equally in one sample and thus generate a possible symmetrical pattern. In the bending test, the test specimen was firmly attached at the sprue‐adjacent side. At the free end, 0.5 kg of weight was added to the specimen with each image capture. In the unloaded state, no colors are visible due to tempering. With the first level of loading, the isochromatic lines along the stress direction become visible. With increasing load, the isochromatic lines increase at the edge of the specimen. Again, these lines accumulate at the edge. Additionally, Figure 9 illustrates the sequence of color changes, progressing from orange to red, then to blue, and finally to green.

**FIGURE 10:**
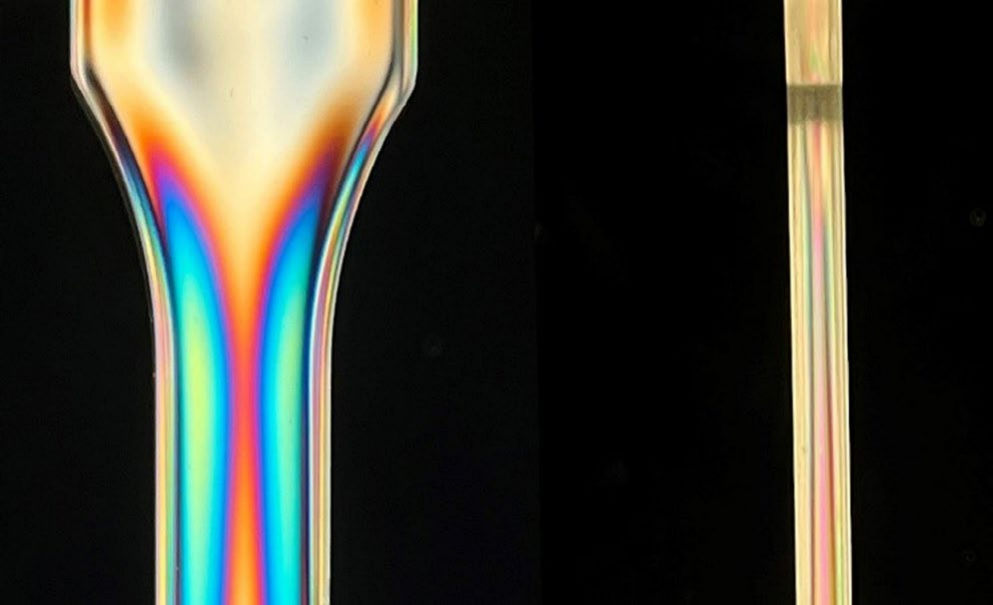
Close‐up of the isochromatic fringe patterns in test specimen (mold 1, variation 1) without external load.

To further correlate the polariscope images with the color scale obtained from Figures 9 and 10 provides a closer examination of the edge region. Clearly visible are the repeating colors at the edge, which appear symmetrically. This confirms the assumption that the geometry used, in conjunction with the injection molding process settings, leads to residual stress in the edge layer of the specimens (near the sprue). Upon closer inspection, the previously described effect occurs: The higher the complete wavelength reputation, the more the color intensity diminishes, causing the edge to appear white; this complicates the recognition of the isochromatic fringe patterns and the analyses of the residual stress state. The side view of the samples highlights this effect even more due to their smaller dimensions. Due to these uncertainties and lack of quantitative values of the residual stress state with the polariscope, the hole drilling method was also employed.

### 3.3 Differential Scanning Calorimetry (DSC)

DSC measurements were carried out to investigate the morphology of different areas (edge and core, see Figure 4) for test specimens produced by both mold tools. For mold tool 1, the test specimen was variation 1, and for mold tool 2, variation 7 was chosen.

The injection molding‐related differences in crystallinity in core and edge areas and the occurrence of an amorphous boundary layer lead to deviating material properties throughout the cross‐section of the test specimen [38]. The difference between the considered edge and core areas at the three positions of the sample extraction from the test specimen is shown again schematically in Figure 4 (right), where the amorphous surface is highlighted blue, and was investigated in previous work [32]. The edge area has a theoretically larger proportion of amorphous material due to the edge surface, while the core area is prepared from the core of the specimen with no amorphous surface area.

**FIGURE 11:**
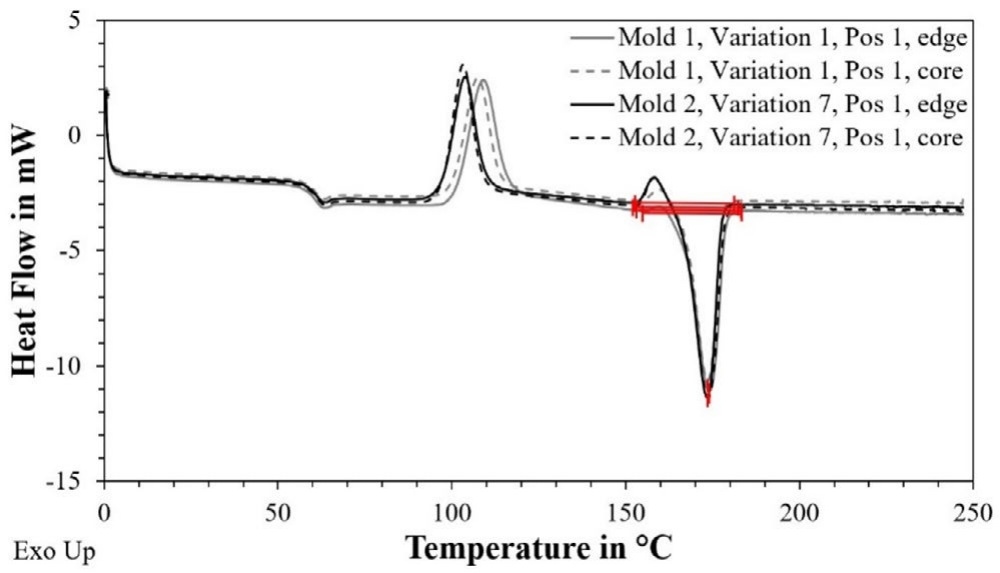
Exemplary graphs of the DSC analysis for the characterized samples at the edge and core area of position 1 (see Figure 10) of the test specimens produced with mold 1, variation 1 and mold 2, variation 7.

Figure 11 shows the curves (heat flow over temperature) of the DSC measurements in the edge and core area of position 1 of the two samples from mold 1, var. 1, and mold var. 7. It is obvious that the curves only differ in the temperature range of approx. 90°C–120°C Since this peak represents the enthalpy for crystallization (Hc), different high and wide peaks here indicate different degrees of crystallization in the samples. The smaller the peak, the lower the (re)crystallization due to a higher degree of crystallization (Xc). In general, based on these results and the crystallization peak in the range between 90°C and 120°C, it can be seen that annealing the test specimens at 60°C for 60 min is very unlikely to have led to recrystallization.

The range of the melt peak in the temperature range between approx. 150°C and 190°C, in which the enthalpy for calculating the crystallinity was also determined, hardly differs between the four measurements, which indicates very similar values of crystallinity. The very similar curves of the two samples in the DSC analysis, which indicate a similar degree of crystallinity in the material, can be a basis and justification for the almost identical stress–elongation curves of the two samples in Figure 8.

The observation of these two measurement regions was important, as the path traveled by the light in the front and side view was not the same due to the component geometry. This meant that it was not possible to determine with complete certainty whether the residual stress state on the front and side views was similar, as the polariscope samples could not be compared with each other due to the different geometric dimensions. Since it was not possible to measure the residual stress on the side of the specimen due to the limited space available, it was crucial to provide information about the similarity in the crystallinity value of the surfaces. The crystallinity values of the individual measurements are shown in Table 4. Also shown is the standard deviation for both molds in the edge area, as well as from the core area of the test specimen for the measurement positions near the sprue (Pos. 1), in the core (Pos. 2) and far from the sprue (Pos. 3). By examining the morphology at these measuring points, the results initially showed that the crystallinity value in each of the three positions of the edge and core areas is very similar for both mold tools. In addition, it can be seen that the degree of crystallization for both mold tools is higher in the core of the specimens, which can be attributed to the longer high temperatures present in the core during the injection molding process and the associated longer time for crystallization.

**TABLE 4:** Crystallinity values (Xc [%]) of the samples shown in Figure 9. Measured in the edge and core areas of samples.

Mold 1, variation 1	Edge area		
	Pos. 1	Pos. 2	Pos. 3
	8.8 ± 0.5	9.0 ± 0.05	9.1 ± 0.2
	Core area		
	Pos. 1	Pos. 2	Pos. 3
	9.8 ± 0.2	10.1 ± 0.7	9.5 ± 0.2
Mold 2, variation 7	Edge area		
	Pos. 1	Pos. 2	Pos. 3
	10.2 ± 0.04	10.8 ± 0.2	10.8 ± 0.3
	Core area		
	Pos. 1	Pos. 2	Pos. 3
	10.8 ± 0.7	10.9 ± 0.4	11.2 ± 0.1

Based on these results, no direct correlation can be made between the apparent isochromatic fringe patterns near the sprue in the polariscope and the crystallinity value.

The calculation of the crystallinity was extended by measurements on the test specimens shown in Figure 2 in order to investigate whether, in addition to the local differences in the edge and core of the test specimen, there are also differences within the various process variations investigated. For this purpose, measurements were carried out similar to the positions in Figure 4b, that is, at the edge area near the sprue and opposite the sprue. The crystallinity values in correlation to the process variations are summarized in Table 5. The results of test specimens a to e are in agreement with the test specimens in Figure 2a–e. The results show that mold tool 1 as well as tool 2 and the annealed test specimen exhibit very similar crystallinity values.

**TABLE 5:** Crystallinity values of the samples shown in Figure 2a–e in correlation to the injection molding process parameters. Measured from the edge area near the sprue and far from the sprue.

	Specimen a	Specimen b	Specimen c	Specimen d	Specimen e
Mold	1	1	1	2	2
Variation	1	2	1	6	7
Annealing	—	—	Annealed	—	—
Near sprue	10.9 ± 0.1	11.8 ± 1.2	9.6 ± 0.9	11.4 ± 0.6	10 ± 0.1
Opposite sprue	9.86 ± 1.5	13.4 ± 0.5	7.7 ± 1.1	11.1 ± 0.4	11.2 ± 0.2

### 3.4 Hole Drilling Method

**FIGURE 12:**
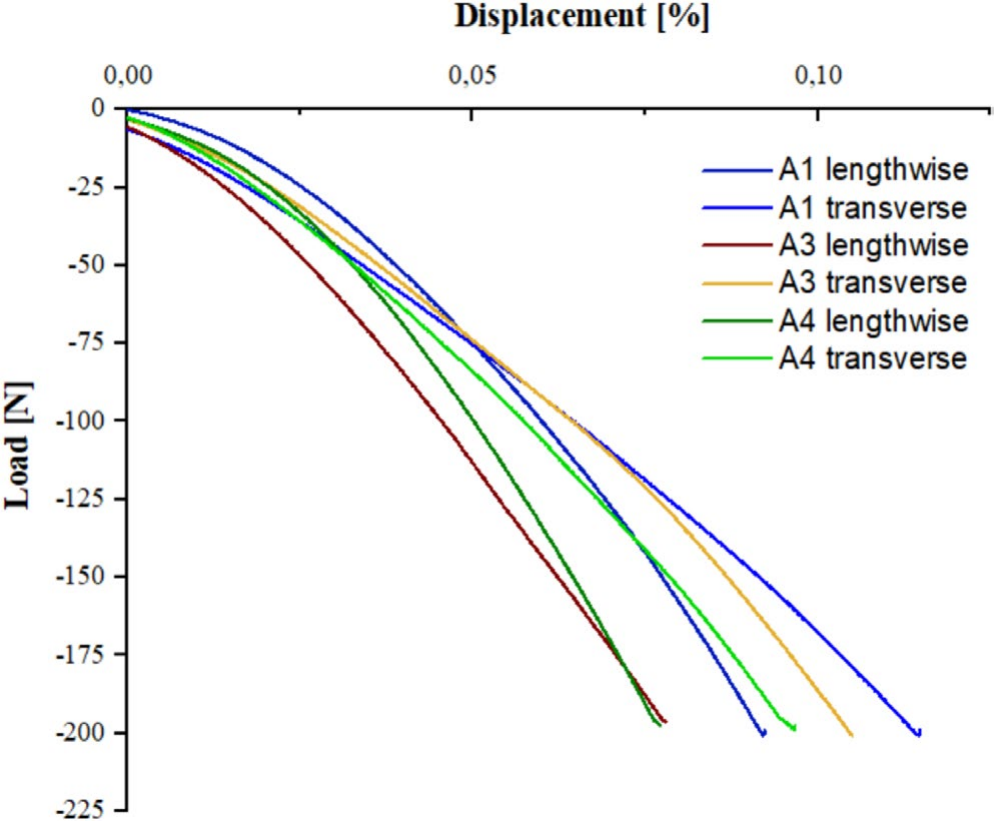
Compression test with measured load–displacement ratio for isotropy measurement of PLA.

The results of the compression tests, conducted to provide for an assessment of isotropy, can be seen in Figure 12. The direction named lengthwise is parallel to the long side of the test specimen, while the direction named transverse is perpendicular to that. The experiment reveals a difference in terms of the mechanical properties in both directions, that is, displacement values obtained at the highest load differ by about 5%. This value of deviation allows for the performance of residual stress measurements applying the hole drilling method according to the ASTM standard.

The measuring strategy applied comprised the detection of “hotspots” with the help of the polariscope, that is, the areas with the highest density of isochromatic fringe patterns. This procedure finally allowed analyzing many test specimens with different color patterns in the most efficient manner. After localizing hot‐spots as well as fully transparent areas, the strain gauges were glued to the middle of the shoulder as a reference point (Figure 6). To obtain first results, five specimens with different color patterns under polarized light were chosen. For each mold tool, one random test specimen characterized by an average residual stress state, as revealed by minor color changes under polarized light (mold 1, variation 1 and mold 2, variation 7), and one test specimen showing almost no color (mold 1, variation 2 and mold 2, variation 6) were chosen. For reference, one specimen that had been annealed to reduce residual stress (mold 1, variation 1, annealed) was analyzed.

**FIGURE 13:**
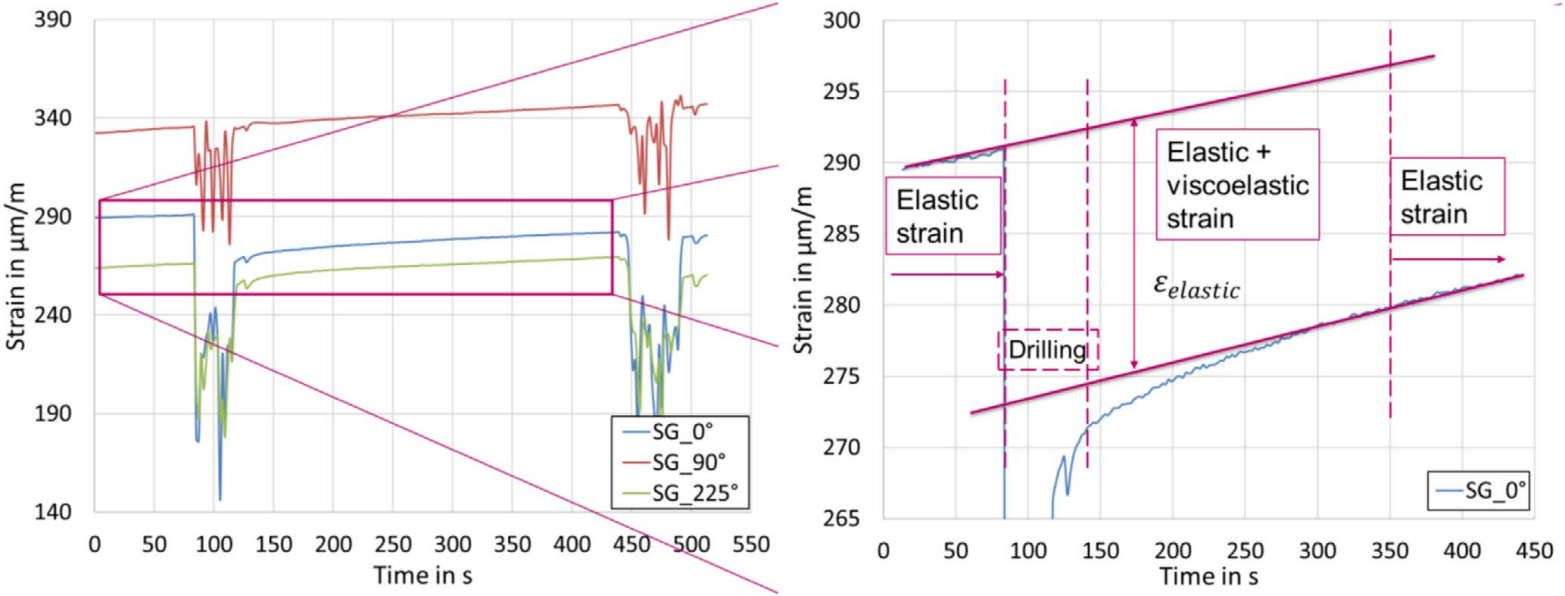
Strain evolution influenced by creep and the drilling process. Interval between drilling = 5 min.

Since mostly all polymers have a tendency to creep over time, direct usage of strain data would not be representative, since it includes released strain with a combination of creep. To reduce the interference of creep, the derivation of obtained strain was used, as it could be seen in Figure 13 [26].

To define measurement accuracy, the standard errors in the combination strains were estimated [30]. Since measurement error lies below 10^−9^ (μm/m)^2^, we calculate the error for calculated residual stresses by adjusting acquiring time for strain data.

**FIGURE 14:**
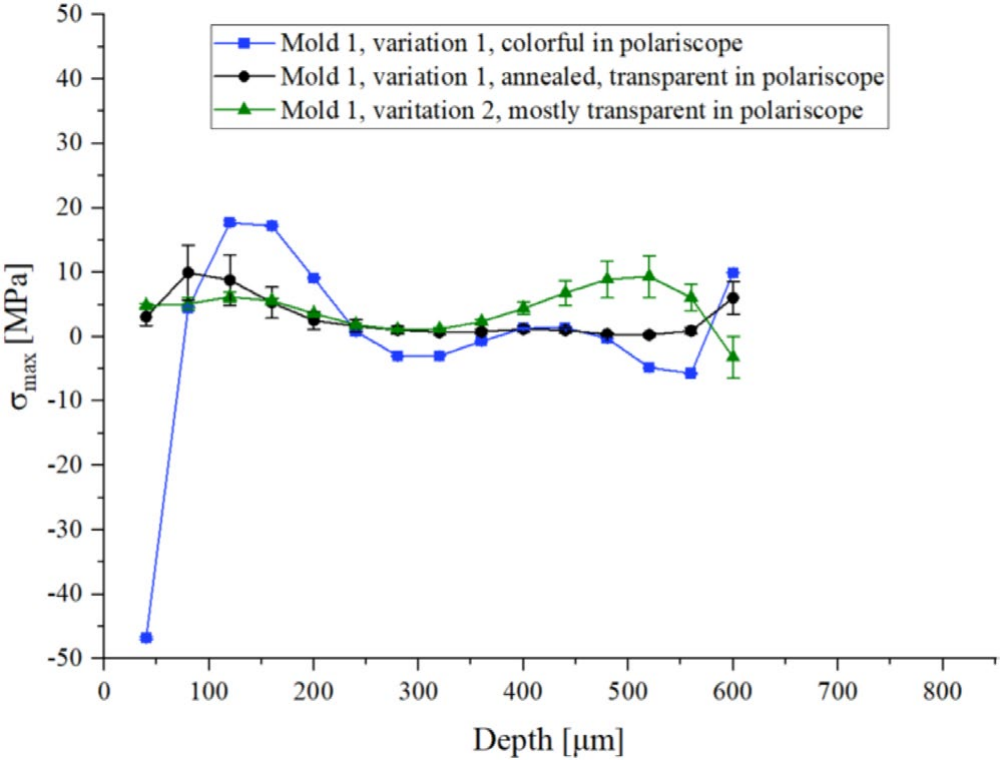
Residual stress profile with error bar of specimens with different color distributions produced by the ejector pin mold tool.

As predicted, the isochromatic fringe patterns can be directly correlated to the residual stress distribution. In Figure 14, the blue curve shows the depth profile of residual stress in the colorful segment of the test specimen produced with mold 1 and variation 1. In contrast, the area with hardly visible fringe patterns of the specimen processed with mold 1 and process variation 2 is characterized by minimal residual stress values (green curve). Accordingly, the colorless area of the subsequently annealed sample (mold 1, variation1, annealed) shows almost a zero value for the residual stress in the entire depth profile (black curve).

**FIGURE 15:**
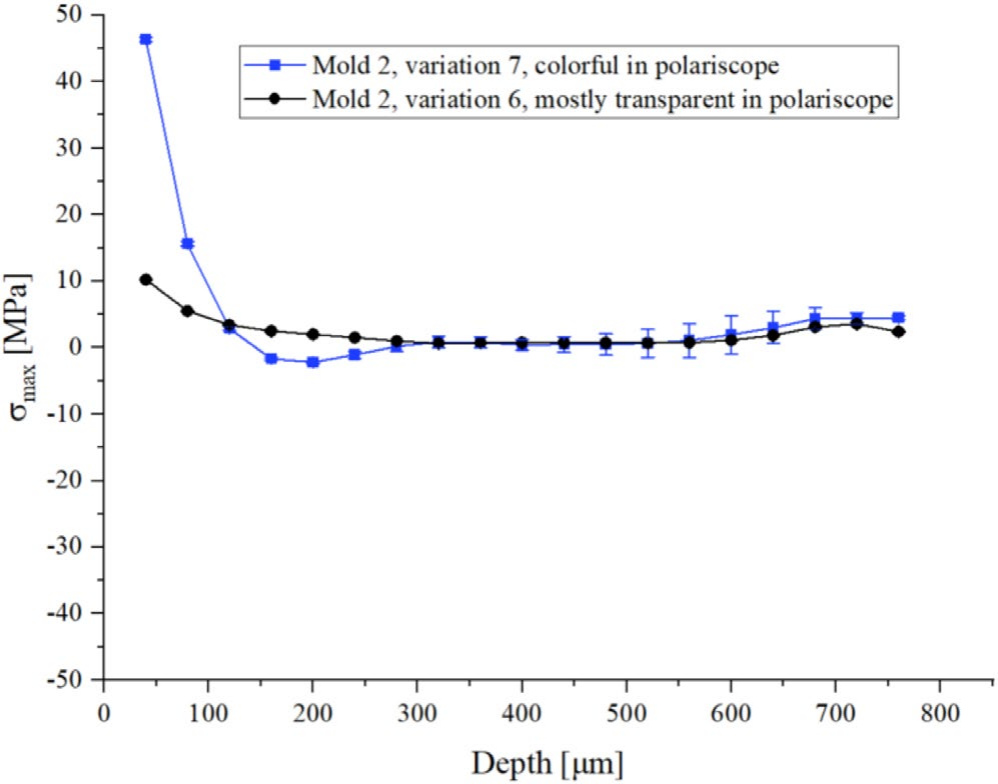
Residual stress profile with an error bar of specimens with different color distributions produced by the full‐surface ejector mold tool.

To confirm the results from the previous experiments and to finally check the initial assumption, more specimens were produced using mold 2 (full‐surface ejector). As can be seen in Figure 15, a clear correlation between the isochromatic fringe patterns and the magnitude of residual stress can be seen. This could be confirmed by the test specimen processed with mold 2 and process variation 6, showing hardly any isochromatic fringe patterns, which also shows an almost stress‐free state according to the results obtained by the hole drilling method (black curve).

It is important to note at this point that the specimen produced with the full‐surface ejector mold tool (mold 2) shows a tendency to produce parts with tensile stresses on the surface, while the specimen of the ejector pin mold tool (mold 1) shows compressive stresses on the surface (Figure 15).

The specimens from both molds show a residual stress profile with the highest stress values, be it compression or tension, at the surface. In depth, a transition to opposite signs can be seen within the range of drill depth, which is characteristic of a residual stress profile with symmetrical distribution.

After quantitative evaluation of residual stress profiles through the hole drilling method following the initial assessment of the isochromatic fringe patterns, the following conclusions can be drawn: The geometry of molding tools leads to different kinds of correlation between the isochromatic fringe patterns and the residual stress distribution. Since polariscope analysis provides information throughout all depths, it cannot differentiate between the residual stress on the surface or in depth, that is, the appearance of the color distribution can reflect the magnitude of residual stress, but not the actual sign of the stress prevailing.

## 4 Discussion

In the present study, test specimens with different residual stress states were successfully produced using two different mold tools. It was observed that PLA responds to varying machine settings with structural changes, resulting in different residual stress distributions. With the polariscope, residual stress in the edge area could be clearly localized. These isochromatic fringe patterns appeared symmetrically within the test specimens. This is typical for polymers and occurs during the injection molding process and its different cooling conditions [15, 28].

To determine the residual stress distribution, the similar approach of using analysis of isochromatic fringe patterns was suggested in [21]. In this study, a more conventional approach to quantitatively measure the residual stress distribution in depth was used to determine the dependence of the residual stress state on the color pattern. The standardized measurement technique provides the basis for a better understanding of the correlation between process parameters and residual stress distribution using isochromatic fringe pattern analyses. Nevertheless, the experimental results from [21] show similarities in the stress distribution on the surface; in particular, the highest amount of residual stress is observed in the shoulder region.

**FIGURE 16:**
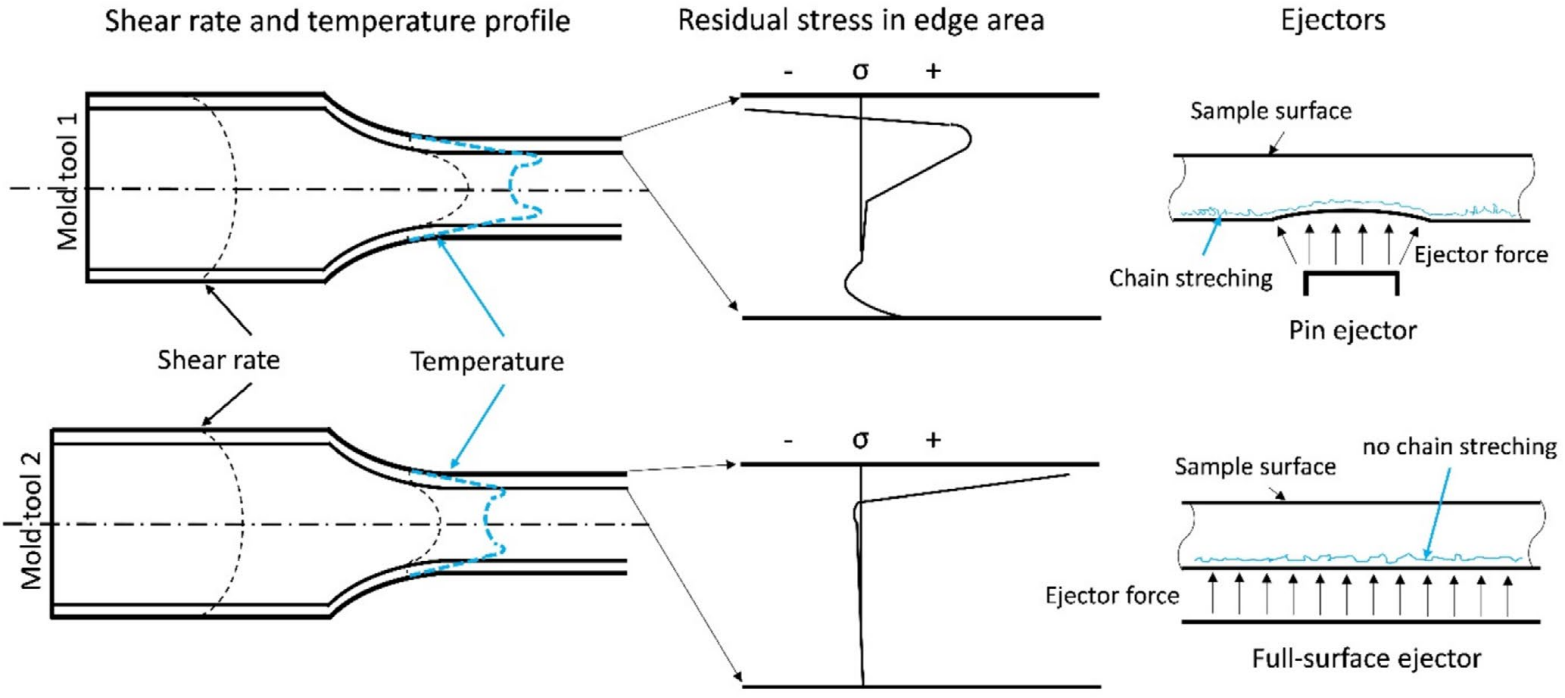
Shear rate and temperature profile of both mold tools with corresponding residual stress distribution and the schematic ejector forces.

The hole drilling method was used to validate and quantify the residual stress values at significant local points and corresponded them with the polariscope images. Each mold tool resulted in different residual stress states within the test specimens. However, the area with the highest residual stress values was consistently in all test specimens between the edge and the core. With one exception (mold 1, variation 2), the hole drilling method always showed the highest residual stress on the surface. The magnitude and nature of the stress differed, as schematically illustrated in Figure 16. For instance, with ejector pins, a stress range from −30 MPa to 25 MPa was measured. As shown in Figure 16, the values transition from compressive to tensile stress. The results of the residual stress from the full‐surface mold tool range from nearly 50 MPa in the tensile region to almost 0 MPa.

Literature often suggests that the polymer melt in the core cools down later than in the outer layers, leading to tensile stress inside and compressive stress on the surface. However, for PLA, which is a semi‐crystalline polymer, this assumption cannot be fully made without considering crystallinity. In the investigations of crystallinity in this work, which were carried out using DSC measurements, the results showed no clear correlation between occurring colors made by the polariscope and the crystallinity value. Depending on the manufacturing conditions, different thicknesses of amorphous outer layers can be formed [28, 32]. This amorphous layer is not ideally uniform across the entire component, which can be explained by the surface roughness of the tool [12]. In the case of the used test specimens, the thicknesses of theses layers vary from 30 to 110 μm. The outer layers are influenced by the temperatures and pressures during injection molding. The fact that the polariscope images show a color development in the edge area and thus the highest residual stress prevails there, as well as the DSC measurements have shown an equal morphological composition of the material both edge and core, suggest that a measurement up to 600 to 800 μm is sufficient to localize the highest residual stresses. Since the present experiments drilled to a such a depth, this covers the area of highest color intensity in the polariscope images. It can be assumed that further experiments with deeper drilling will not yield higher stress values.

The annealing of the samples from mold 1, variation 1 did not lead to an increase in crystallization, which can be seen from the DSC measurements but also from the lack of opacity of the test specimen after annealing. This is due to the low temperature during annealing. Nevertheless, the annealed test specimens show almost no color in the polariscope and therefore no residual stresses. This suggests that the temperature and time during annealing were sufficiently high to relieve stresses introduced by the cooling conditions in the injection molding process, for example.

Comparing the residual stress states of both mold tools reveals different edge stress distributions. The ejector pin mold tool exhibits a residual stress distribution arising from thermal and cooling conditions [14]. Full‐surface mold tool, on the other hand, shows a residual stress distribution due to flow conditions [14]. Besides the ejectors, the main difference between the two mold tools is the varying shoulder length. Due to the shorter melt path of the ejector pin mold, the shear rate in the tapering section is high. In this complex process, multiple temperatures play a role in the formation of the outer layer. This involves the temperature difference between the cool mold tool and the melt, as well as the temperature in the shear rate area between the solidified melt at the edge and the flowing melt. These factors are the reasons for the development of residual stress, explaining the high residual stress in the outer layer and the sprue area. The full‐surface mold tool has a longer inflow path, resulting in lower shear rates and smaller temperature gradients. In addition to this longer inflow path, it differs by using full‐surface ejectors. Since the causes of residual stress in a component stem from both thermal and mechanical origins, the choice of ejectors plays a role [13]. The geometry and design of ejectors can significantly impact the surface of the component [39]. Moreover, the ejection process can lead to shrinkage due to deformation induced by the ejector force [40]. As schematically illustrated in Figure 16, the ejector pin can cause dimpling of the surface. In extreme cases, for example, if the sample has not cooled and solidified sufficiently, this can lead to the elongation or realignment of molecular chains, whereby additional residual stress is introduced into the sample.

## 5 Conclusions

The present study demonstrated how residual stress can manifest to varying degrees even in a structurally simple component and lead to premature fracture failure under external load. The established method of using a polariscope enabled the localization of residual stress. However, the polariscope reaches its limits when dealing with the superposition of colors due to higher orders of refraction. To accurately measure the residual stress quantitatively, the hole drilling method proved to be a suitable tool. Nonetheless, using the hole drilling method is impractical in the rapid production cycle of polymer components because of its measurement duration. Therefore, it is crucial to investigate the correlation between the actual measured residual stress state and the color distribution observed through the polariscope to evaluate potential weak points in the component.

Based on the investigations carried out here, it is clear that residual stresses occurring are not only associated with the crystallinity and the related volume contraction processes during crystallite formation, but can also deviate even with a very similar degree of crystallization. This may be due to stresses caused and influenced by cooling in the tool.

Nevertheless, there is a potential to adapt the combination of both measurement techniques for quality control sequences, where the hole drilling method allows quantification of local residual stress distribution of specific parts, and polariscope examination allows quick investigation of the whole specimen.

## Author Contributions

Conceptualization: M.R. and O.T. Methodology: M.R. and O.T. Formal analysis: M.R. and O.T. Investigation: M.R. and O.T. Resources: M.R. and O.T. Data curation: M.R. and O.T. Writing – original draft preparation: M.R. and O.T. Writing – review and editing: J.‐C.Z. and A.L. Visualization: M.R. and O.T. Supervision: H.‐P.H. and T.N. Project administration: H.‐P.H. and T.N. All authors have read and agreed to the published version of the manuscript.

## Conflicts of Interest

The authors declare no conflicts of interest.

## Data Availability

The data presented in this study are available on request from the corresponding author.
